# Socioeconomic Gradients in Different Types of Tobacco Use in India: Evidence from Global Adult Tobacco Survey 2009-10

**DOI:** 10.1155/2015/837804

**Published:** 2015-07-26

**Authors:** Ankur Singh, Monika Arora, Dallas R. English, Manu R. Mathur

**Affiliations:** ^1^Department of Health Promotion, Public Health Foundation of India, Gurgaon, Haryana, India; ^2^Australian Research Center for Population Oral Health (ARCPOH), University of Adelaide, Adelaide, SA 5000, Australia; ^3^Melbourne School of Population and Global Health, University of Melbourne, Melbourne, VIC 3010, Australia

## Abstract

Socioeconomic differences in tobacco use have been reported, but there is a lack of evidence on how they vary according to types of tobacco use. This study explored socioeconomic differences associated with cigarette, bidi, smokeless tobacco (SLT), and dual use (smoking and smokeless tobacco use) in India and tested whether these differences vary by gender and residential area. Secondary analysis of Global Adult Tobacco Survey (GATS) 2009-10 (*n* = 69,296) was conducted. The primary outcomes were self-reported cigarette, bidi smoking, SLT, and dual use. The main explanatory variables were wealth, education, and occupation. Associations were assessed using multinomial logistic regressions. 69,030 adults participated in the study. Positive association was observed between wealth and prevalence of cigarette smoking while inverse associations were observed for bidi smoking, SLT, and dual use after adjustment for potential confounders. Inverse associations with education were observed for all four types after adjusting for confounders. Significant interactions were observed for gender and area in the association between cigarette, bidi, and smokeless tobacco use with wealth and education. The probability of cigarette smoking was higher for wealthier individuals while the probability of bidi smoking, smokeless tobacco use, and dual use was higher for those with lesser wealth and education.

## 1. Introduction

Mortality and morbidity due to active smoking and the resulting involuntary exposure of nonsmokers to tobacco smoke are well substantiated globally [1–3] and in India [4–8]. While a recent multicountry study reported global reductions in cigarette smoking [9], the Indian Global Adult Tobacco Survey [10] reported high smokeless tobacco (SLT) use among both men and women. Considering the availability of tobacco in myriad varieties in India in addition to smoked forms of tobacco, cigarettes and bidis (tobacco rolled in a leaf), it is complicated to assess the overall tobacco burden in India [11]. The growing burden of noncommunicable diseases (NCDs) associated with tobacco use in India points towards the need to study its underlying determinants in order to design appropriate policy interventions to address this public health issue.

Previous studies have also assessed and reported socioeconomic differences in tobacco use both globally [12–17] and in India [18–21]. A study conducted by Thakur et al., 2013, revealed differences according to geographical regions in the association between socioeconomic attributes with smoking and smokeless tobacco use. The study further revealed consistent inverse gradients for both smoking and smokeless tobacco use in India [22]. On the contrary, a recent study conducted by Corsi and Subramanian (2014) assessed socioeconomic inequalities in smoking behavior amongst males in India and reported that while cigarette smoking was concentrated among people who were wealthier, more educated, and with higher occupational status, on the contrary bidi smoking was more concentrated among the disadvantaged [19]. Similar contrasting gradients have also been reported from a regional study in India [23]. This unusual variation in socioeconomic gradients in consumption of the two smoking products among Indian males raises both concerns and curiosity to assess how usage across the different types of tobacco products (SLT and cigarette, bidi) differs by socioeconomic profile. While this inconsistency in results highlights the importance of treating each of these types of tobacco products differently, a greater concern which has been ignored in these studies is the growing prevalence of dual use (use of both smokeless and smoking forms of tobacco) in India [24]. Dual users are potentially at a greater risk for morbidity and mortality when compared with those who use one tobacco product only [25].

Most of the previous studies from India reported socioeconomic differences in tobacco use but to our knowledge none has studied the socioeconomic differences in tobacco use for all the different types of tobacco collectively or assessed the variations in these differences according to gender and area of residence. To address this gap in evidence, we therefore assessed the socioeconomic differences in different types of tobacco use (smoking (cigarette, bidi), SLT, and dual tobacco use) in India and further studied the variations in some of these differences according to gender and area of residence using a nationally representative survey of tobacco use in India.

## 2. Methodology

### 2.1. Study Population

The Global Adult Tobacco Survey (GATS 2009-2010) is a multicountry household survey launched in 2007 for formulation, tracking, and implementation of effective tobacco control interventions in the study countries. We analyzed data from 69,296 adults (ages 15 years and above) from the Indian GATS, which was conducted in 2009-10. The sample was drawn using multistage sampling. In urban areas, the primary sampling units (PSUs) were the city wards, the secondary sampling units (SSUs) were the census enumeration blocks, and the tertiary sampling units (TSUs) were households. In rural areas, villages comprised the PSUs [10].

### 2.2. Eligibility Criteria

Individuals aged over 15 years in the identified PSUs and living in the selected households were eligible to participate in the survey. All noninstitutionalized individuals who gave their agreement to voluntarily participate in the study were eligible. In the case of minor respondents (15–17 years), consent was sought from the participant as well as from their parent/guardian [10].

### 2.3. Variables

GATS data was collected using household and individual questionnaires that were developed in English and later translated into 19 regional languages [10]. The self-administered individual questionnaires covered information broadly on the following eight sections: demographic characteristics, tobacco smoking, SLT use, cessation, second hand smoke, economics, media and knowledge, and attitude and perceptions. Details of the sampling procedure and data collection have been published [10].

The primary outcomes for this analysis were self-reported current smoking and SLT use. Respondents were asked, “On average, how many of the following products do you currently smoke each day? Also, let me know if you smoke the product, but not every day.” Those who responded smoking one or more than one for manufactured/rolled tobacco in paper and leaf daily were categorized as current cigarette smokers and those who responded smoking one or more than one bidi were categorized as current bidi smokers. For the outcome of current SLT use the respondents were asked, “Do you currently use smokeless tobacco on a daily basis, less than daily, or not at all?” All those who answered “daily” or “less than daily” were recategorized as “Yes” and those who responded “not at all” and “do not know” and “refused” were recategorized as “No” considering that there were no observations in these categories. Those respondents who answered yes to both current smoking (cigarette, bidi smoking) and current SLT use were categorized as dual users. In order to avoid duplication of these respondents in current smoking (cigarette, bidi smokers) and current SLT users, these respondents were excluded from only cigarette, bidi, and SLT users in previous categories. Hence, the four outcomes were exclusive cigarette smoking, bidi smoking, smokeless tobacco use, and dual users.

Socioeconomic status, the main explanatory variable, was assessed through “educational attainment,” “wealth,” and occupational groups. Educational attainment, measured through the “highest level of education completed,” was categorized as “no education,” “primary school or less,” “less than secondary school,” and “more than secondary school.” Principal components analysis (PCA) of household assets was used to create a wealth index [15]. Assets included electricity, flush toilet, car/scooter, motorcycle, television, refrigerator, washing machine, telephone and mobile phone, and radio. The wealth index was divided into quintiles. The occupational groups were categorized as “government employee,” “private employee,” “housewives, students, and retirees,” “unemployed but able,” and “unemployed and unable.” Respondents with missing information on education, wealth, and occupation were excluded from the analyses [10].

Other covariates included age, sex, area of residence (urban versus rural), and geographical region of India. Analysis adjusted for age (measured in years) was categorized using six groups: “15–17” (minors), “18–30,” “31–45,” “46–60,” “61–75,” and “76 and above”.

### 2.4. Statistical Analysis

Multinomial logistic regression was used to estimate odds ratios and attendant 95% confidence intervals for the associations between tobacco use and socioeconomic variables (education, wealth, and occupation). Multinomial logit model (MNLM) simultaneously allows estimation of binary logits for all possible comparisons among different outcome categories and is well suited to examine multiple outcomes [26]. In order to conduct this regression, a composite nominal variable with nonusers as the reference and cigarette, bidi, SLT, and dual users as index categories was created and regression models were fitted with each of the SES variables.

In the first models, the outcomes were fitted with each SES variable alone (Model 1). Demographic variables of age, sex, area of residence, and geographical regions were included in the next set of models (Model 2). Finally, Model 3 included these demographic variables and all SES variables simultaneously. Model 3 was extended by fitting interactions (one at a time) between the socioeconomic variables (wealth and education) and gender and place of residence. Participants who reported dual use (5.3%) were dropped from the interaction analyses. We further tested for differences in the wealth and educational gradients between the different tobacco products. We accounted for the sampling design and the sample weights [27] by using the “survey” command in Stata, version 11.1 (StataCorp, College Station: TX). All *p*-values reported are from Wald's tests.

## 3. Results

Overall 69,296 respondents participated in the GATS with a response fraction of 91.8% (GATS, 2010). We excluded 266 respondents (0.038%) who did not report socioeconomic status (SES) information, leaving 69,030 respondents for the analysis. The sociodemographic profile of the participants is described in Table 1. About half of the sample were male, almost half were 15–30 years of age, 70% were from rural areas, and 31% had no formal education.

The prevalence of current SLT use (20.5%) was much higher than the prevalence of cigarette smoking (2.8%), bidi smoking (5.7%), and dual use (5.3%) (Table 1). These differences were more pronounced for females than for males and in rural compared with urban areas. Compared with other tobacco products, use of smokeless tobacco was much more prevalent among 15–17 year olds. The prevalence of current SLT use varied significantly with educational attainment and wealth. While SLT use, bidi smoking, and dual use were inversely associated with wealth, cigarette smoking was positively associated with wealth. Similarly, the prevalence of current cigarette smoking was positively associated with education while prevalence of SLT use was inversely associated with education. Compared with other occupational groups, homemakers, students, and retirees had much lower prevalence for any type of tobacco use (Table 1).

Table 2 shows results of the multinomial logistic regression analyses. Wealth was positively associated with cigarette smoking both crudely and after adjustment for demographic factors. The association became stronger after adjusting for educational attainment and occupation. The odds ratio for the richest category was 3.86 (95% CI: 2.54–5.86) relative to the poorest group. Bidi smoking, SLT use, and dual use were inversely associated with wealth after adjusting for demographic variables as well as education and occupation. For bidi smoking, after adjusting for education and occupation the odds ratio among poorer groups compared to the richer groups became closer to one while the association between SLT use and dual use with wealth changed a little after adjustment for educational attainment and occupation (Table 2).

Similar to the association between cigarette smoking and wealth, cigarette smoking was positively associated with educational attainment in the unadjusted analysis (Model 1). Without adjustment for wealth, educational attainment was not associated with cigarette smoking (Model 2), but after adjustment, it was inversely related (Model 3). Educational attainment was inversely related to bidi smoking, SLT use, and dual tobacco use. Of the four types of tobacco use, bidi smoking had the strongest association with education after adjustment for wealth and occupation (Table 2).

Government employees had the highest odds ratio for cigarette smoking 3.27 (1.34, 7.99), nongovernment employees had the highest odds ratio 2.00 (1.36, 2.96) for bidi smoking, and self-employed had the highest odds ratio for SLT use 1.60 (1.26, 2.03) compared with those who were unemployed and unable to work. The highest odds for dual use was observed for those unemployed and able to work 2.56 (1.44, 4.54) when compared with those who were unemployed and unable to work (Table 2). All *p*-values comparing the coefficients for wealth and educational attainment for the different types of tobacco were < 0.001.

**Figure 1:**
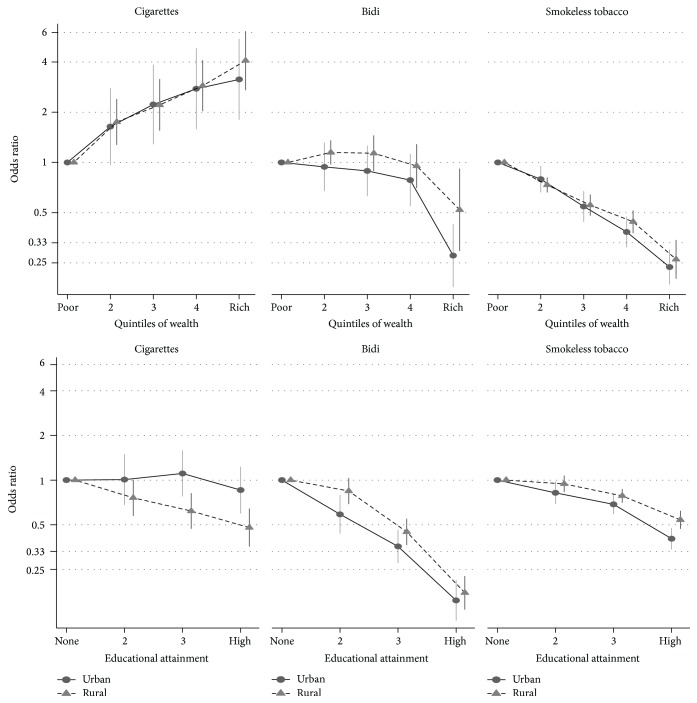
Urban-rural differences in educational and wealth gradients in the relationship between prevalence of cigarette smoking, bidi smoking, and smokeless tobacco use and socioeconomic status in India (odds ratios adjusted for age, gender, area of residence, and education and wealth).

The positive association between cigarette smoking and wealth did not vary by region (*p* interaction = 0.88, Figure 1), while for education there was no association in urban areas but an inverse association in rural areas (*p* = 0.03, Figure 1). For bidi smoking and SLT use, urban and rural regions had similar inverse associations with wealth (bidi, *p* interaction = 0.23; SLT, *p* = 0.80) and education (bidi, *p* interaction = 0.05; SLT, *p* = 0.09).

**Figure 2:**
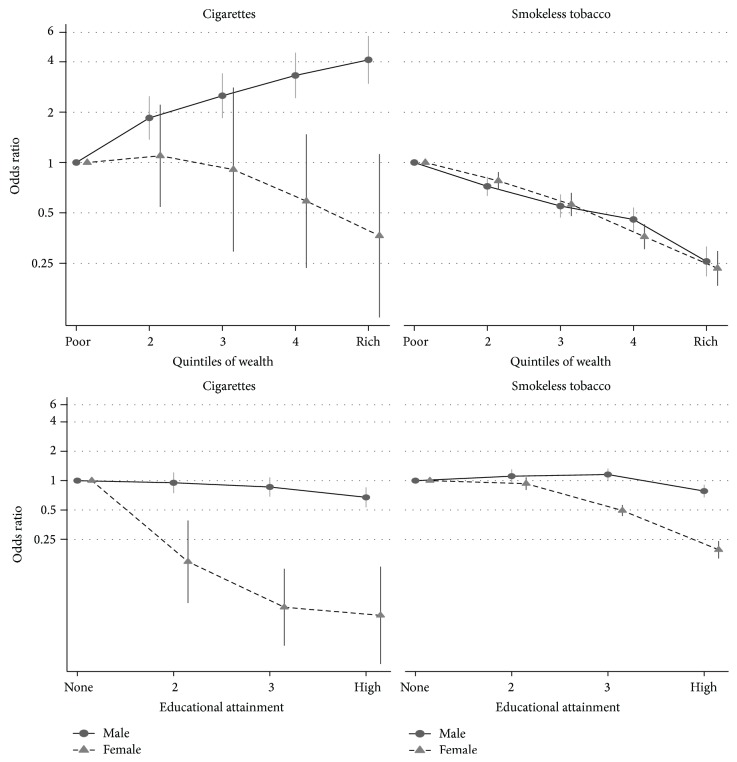
Gender differences in educational and wealth gradients in odds for association between cigarette and smokeless tobacco with socioeconomic status in India (odds ratios adjusted for age, gender, area of residence, and education and wealth).

While a positive association was observed between cigarette smoking and wealth for males, an inverse association was observed for females (*p* interaction = 0.0017, Figure 2). For males, there was little association between cigarette smoking and education, but a strong inverse association for females (*p* interaction < 0.0001, Figure 2). For SLT, males and females had similar inverse associations with wealth (*p* interaction = 0.38, Figure 2), but the inverse association with education was stronger for females (*p* interaction < 0.0001, Figure 2). Too few women smoked bidi smoking to test interactions between SES and gender for this outcome.

## 4. Discussion

The current study assessed associations of current tobacco use with socioeconomic positions and further studied gender and area wise differences using a nationally representative sample from India. Marked socioeconomic differences in the most prevalent forms of tobacco use (cigarettes, bidi, SLT, and dual use) were observed. While cigarette smoking had positive associations with wealth, inverse associations were observed for bidi smoking, SLT use, and dual use. Consistent positive associations were observed with educational attainment for all three forms of tobacco use and variations were observed in the probability of different types of tobacco use according to different occupational groups. With regard to wealth, bidi smoking showed larger variation according to area of residence when compared with cigarette smoking and SLT use regardless of the direction of the association. Considerable variations according to gender in the socioeconomic (both wealth and education) gradients were observed for cigarette smoking.

Several studies have previously assessed and identified the importance of social determinants of tobacco use both globally and in India [6, 9, 11, 13, 15, 17–19, 28–30]. Of the studies which assessed these inequalities in India, some assessed the socioeconomic differences at a multicountry level [13, 15] while others reported inequalities at national [2, 20, 21] and subnational level [30]. A previous study from India based on data from earlier surveys reported that India is currently between stages II and III of the cigarette epidemic model only for men, but it distinctly differs from the model on the patterns observed for women [18]. Based on the findings of the current study we also observe that it is difficult to classify tobacco use in India under the conventional cigarette epidemic model due to the considerable variations in the socioeconomic gradients by different types of tobacco use.

Consistent with findings of previous studies [19, 23], the current study also observed divergent gradients for cigarette and bidi smoking. The current study further substantiates these findings by showing that, apart from bidi smoking, the SLT and dual use also follow the same pattern. Hence, an obvious interpretation of these findings is that tobacco usage in the Indian subcontinent is very different from that in high income countries as there is ample evidence on social gradients in cigarette smoking from high income countries suggesting higher prevalence of smoking among lesser educated and income groups [31, 32] while these gradients differ according to tobacco products in India and surrounding countries. To some extent this shows that higher disposable income along with stable occupation (e.g., a government job) are predictors of cigarette smoking but not other types of tobacco use, which is comparatively more prevalent amongst the disadvantaged. The positive association of cigarette smoking and educational attainment was reversed after adjustments for demographic and other socioeconomic variables including wealth and occupation highlighting that educational attainment is a strong predictor for all types of tobacco use in India.

A study conducted by Gupta et al., 2012 [24], showed that while dual use is increasingly becoming a concern for tobacco control in India, few studies have attempted to study its determinants. While the current study reports a low prevalence of dual use in India, the consistent inverse wealth and educational gradients show greater vulnerability of the poorer and lesser educated in comparison with their richer and more educated counterparts. Similarly, considering the strong causal associations reported between SLT use and oral precancerous and cancerous lesions and the increasing evidence of its association with other systemic diseases, the current study also indicates that the inverse wealth and educational gradients may lead to health inequalities in the absence of effective tobacco control policies.

Apart from the evidence reported on geographical variations in the social gradients in smoking and SLT use [22], the current study also found variations according to area of residence and gender. The greater vulnerability of poorer and lesser educated females towards cigarette smoking and SLT use raises important concerns as more health related complications are associated with tobacco use for females when compared with males [33]. Hence, the more disadvantaged females and their families may have to bear a considerable amount of economic burden due to the associated health costs due to tobacco use. These variations in the gradients further point towards the need for future research to study the sociocultural, psychosocial, and material pathways which lead to such health compromising behaviours irrespective of the relative position in the social structure and accordingly frame policies that will reduce demand for tobacco use.

The current study had several strengths and limitations. The study assessed the association of the most prevalent forms of tobacco use with three different measures (wealth, educational-attainment, and occupation). The literature suggests that these measures highlight different underlying socioeconomic processes [34] and the findings from the current study further highlight that different types of tobacco use are associated differently with these socioeconomic attributes. The study also assessed whether these socioeconomic inequalities differ for males and females and also for those living in urban versus rural areas. The current study used multinomial logistic regression, which allowed simultaneous comparisons of different outcome categories. Some limitations of our study could be that the information on tobacco may suffer from social desirability, especially for women as discussed in a previous study [18]. Finally, our analysis of cross-sectional data does not imply causation of these social factors.

The current study has some interesting findings and important research as well as policy implications. The underlying answers to the social inequalities in different types of tobacco use in India cannot be sought without understanding the sociocultural milieu of tobacco use. Future research using more sophisticated measures of social class and social position [35, 36] may help in understanding the relationship between different types of tobacco use and complex socioeconomic processes. The differences in probabilities for types of tobacco use in different occupational groups underscore the need to understand how these employment relations are driving tobacco use in India. The steep socioeconomic gradients in the SLT use compared with cigarette and bidi smoking build evidence for the Ministry of Health and Family Welfare, Government of India's Gutkha (most prevalent form of smokeless tobacco) ban [37], as a whole population approach to reduce the associated public health burden. With the growing prevalence of dual use of tobacco (5.4%) reported by GATS [10] and the current policy scenario (Gutkha ban) future research studies should be designed to study its underlying determinants. Consistent educational gradients across the population further highlight the need to focus on wider determinants of health and point towards the amalgamation of tobacco control activities in school and college education for further reducing the public health burden of tobacco use. The current results in line with WHO's World No Tobacco Day's 2014 theme [38] support the evidence to increase tobacco taxation across all products as a whole population intervention in order to reduce the tobacco use across the social gradients.

## 5. Conclusion

In the light of the differences in social gradients according to types of tobacco use in India the findings from the current study point towards the need to combine tobacco control strategies for the whole population and for targeted or vulnerable subgroups while addressing the underlying determinants or “the causes of the causes” [39].

## Figures and Tables

**Table 1:** Sociodemographic characteristics of the sample according to current tobacco use (*n* = 69,030).

Characteristics	Categories	*n* (%)	Cigarette smoking (%)	Bidi smoking (%)	SLT use (%)	Dual use (%)
Total		69,030 (100)	2,999 (2.8)	4,192 (5.7)	12,668 (20.5)	4,058 (5.3)

Gender	Male	33,685 (51.7)	5.3	9.9	23.6	9.2
	Female	35,345 (48.3)	0.1	1.2	17.2	1.1

Age (years)	15–17	2,878 (7.6)	0.1	0.2	8.3	0.9
	18–30	23,092 (38.4)	1.4	2.2	17.4	4.4
	31–45	25,543 (29.8)	2.7	7.4	23.8	7.0
	46–60	11,758 (16.0)	2.7	11.5	25.3	6.2
	61–75	4,773 (6.7)	1.7	10.8	29.2	5.7
	76 and above	986 (1.5)	0.2	9.1	26.3	8.2

Area of residence	Urban	27,437 (29.3)	4.4	3.7	14.1	3.5
	Rural	41,593 (70.7)	2.2	6.5	23.2	6.0

Geographical regions	North	13,976 (5.2)	4.7	6.6	4.9	2.2
	Central	9,993 (32.5)	1.1	7.2	22.5	6.6
	East	9,686 (21.1)	3.1	5.5	29.8	7.9
	North-East	15,197 (3.6)	4.8	4.2	24.9	9.8
	West	9,091 (14.9)	1.6	3.6	22.4	2.9
	South	11,087 (22.7)	5.1	5.1	10.8	2.6

Educational attainment	No formal education	18,735 (31.0)	1.4	8.3	27.5	6.0
	Less than primary	7,983 (12.2)	2.9	9.4	24.9	8.2
	Primary but less than secondary	19,511 (28.9)	3.2	4.8	19.8	5.4
	Secondary and above	22,801 (28.0)	4.0	2.1	11.7	3.1

Wealth (asset quintiles)	Poorest	13,998 (27.9)	1.1	7.6	30.2	7.8
	Poor	16,033 (26.4)	2.1	6.5	23.0	5.3
	Middle	11,571 (16.5)	3.4	5.8	17.6	4.4
	Rich	13,830 (17.1)	4.4	4.1	13.0	4.0
	Richest	13,597 (12.1)	5.3	1.6	7.7	2.6

Occupation	Unemployed and unable	1,220 (1.9)	1.6	11.9	29.0	5.6
	Unemployed and able	1,500 (2.1)	2.8	5.0	26.8	10.1
	Housewife/retired/student	30,810 (43.2)	0.7	1.7	13.6	1.4
	Self-employed	19,575 (28.5)	4.0	9.9	26.9	8.3
	Nongovernment employee	11,923 (21.1)	4.7	8.0	25.4	8.7
	Government employee	4,002 (3.2)	8.9	4.2	16.4	4.9

**Table 2:** Odds ratios (95% CI) for the association between current tobacco use (smoking, smokeless tobacco, and dual use) and wealth/education (*n* = 67,988).

	Smoking						Smokeless tobacco			Dual use		
	Cigarettes			Bidi								
	Model 1	Model 2	Model 3	Model 1	Model 2	Model 3	Model 1	Model 2	Model 3	Model 1	Model 2	Model 3
*Wealth *												
Poorest	Ref.	Ref.	Ref.	Ref.	Ref.	Ref.	Ref.	Ref.	Ref.	Ref.	Ref.	Ref.
Poor	2.00 (1.35, 2.98)	1.66 (1.11, 2.48)	1.74 (1.16, 2.62)	0.72 (0.63, 0.83)	0.84 (0.72, 0.97)	1.12 (0.95, 1.31)	0.64 (0.58, 0.70)	0.70 (0.64, 0.77)	0.80 (0.73, 0.88)	0.57 (0.48, 0.67)	0.64 (0.54, 0.77)	0.82 (0.68, 0.98)
Middle	3.67 (2.52, 5.34)	2.50 (1.69, 3.68)	2.70 (1.81, 4.05)	0.58 (0.49, 0.70)	0.64 (0.52, 0.78)	1.02 (0.82, 1.26)	0.44 (0.39, 0.50)	0.48 (0.43, 0.55)	0.61 (0.53, 0.69)	0.42 (0.35, 0.51)	0.47 (0.39, 0.58)	0.68 (0.55, 0.84)
Rich	4.94 (3.46, 7.06)	3.14 (2.16, 4.56)	3.38 (2.27, 5.05)	0.38 (0.31, 0.47)	0.41 (0.33, 0.52)	0.81 (0.64, 1.03)	0.30 (0.27, 0.34)	0.32 (0.29, 0.37)	0.44 (0.39, 0.51)	0.35 (0.29, 0.44)	0.39 (0.31, 0.49)	0.65 (0.51, 0.82)
Richest	6.46 (4.52, 9.25)	3.63 (2.49, 5.30)	3.86 (2.54, 5.86)	0.14 (0.10, 0.19)	0.12 (0.08, 0.91)	0.30 (0.21, 0.43)	0.16 (0.14, 0.19)	0.16 (0.14, 0.19)	0.30 (0.21, 0.43)	0.21 (0.16, 0.26)	0.20 (0.16, 0.27)	0.40 (0.30, 0.52)

*Education *												
No formal education	Ref.	Ref.	Ref.	Ref.	Ref.	Ref.	Ref.	Ref.	Ref.	Ref.	Ref.	Ref.
Less than primary	2.56 (1.82, 3.60)	1.14 (0.81, 1.61)	0.97 (0.68, 1.39)	1.15 (0.97, 1.36)	0.69 (0.57, 0.84)	0.75 (0.62, 0.92)	0.92 (0.82, 1.02)	0.79 (0.70, 0.89)	0.92 (0.81, 1.03)	1.39 (1.17, 1.65)	0.81 (0.67, 0.97)	0.89 (0.74, 1.07)
Primary but less than secondary	2.67 (1.99, 3.59)	1.14 (0.85, 1.52)	0.88 (0.64, 1.19)	0.48 (0.41, 0.56)	0.30 (0.25, 0.35)	0.35 (0.29, 0.41)	0.60 (0.54, 0.65)	0.51 (0.47, 0.57)	0.67 (0.61, 0.74)	0.75 (0.64, 0.88)	0.39 (0.33, 0.47)	0.47 (0.40, 0.56)
Secondary and above	4.02 (3.02, 5.34)	1.24 (0.93, 1.65)	0.73 (0.53, 1.02)	0.17 (0.14, 0.21)	0.09 (0.08, 0.11)	0.12 (0.09, 0.14)	0.30 (0.27, 0.33)	0.25 (0.22, 0.28)	0.39 (0.35, 0.44)	0.36 (0.30, 0.43)	0.17 (0.14, 0.20)	0.22 (0.18, 0.27)

*Occupation *												
Unemployed and unable to work	Ref.	Ref.	Ref.	Ref.	Ref.	Ref.	Ref.	Ref.	Ref.	Ref.	Ref.	Ref.
Unemployed and able to work	2.13 (0.86, 5.25)	2.95 (1.15, 7.58)	2.76 (1.07, 7.10)	0.38 (0.24, 0.60)	1.04 (0.62, 1.75)	1.18 (0.69, 2.01)	0.82 (0.62, 1.10)	1.54 (1.13, 2.08)	1.60 (1.17, 2.18)	1.60 (0.95, 2.69)	2.32 (1.32, 4.06)	2.56 (1.44, 4.54)
Housewife, students, and retirees	0.29 (0.12, 0.68)	1.18 (0.49, 2.84)	0.91 (0.38, 2.22)	0.08 (0.06, 0.12)	0.50 (0.33, 0.74)	0.76 (0.51, 1.14)	0.28 (0.22, 0.34)	0.58 (0.46, 0.75)	0.76 (0.59, 0.96)	0.15 (0.10, 0.22)	0.52 (0.33, 0.84)	0.74 (0.46, 1.20)
Self-employed	2.67 (1.18, 6.06)	2.76 (1.17, 6.52)	2.33 (0.99, 5.50)	0.79 (0.59, 1.07)	1.74 (1.22, 2.48)	1.97 (1.37, 2.83)	0.88 (0.71, 1.10)	1.50 (1.18, 1.91)	1.60 (1.26, 2.03)	1.40 (0.94, 2.08)	1.82 (1.17, 2.82)	2.02 (1.30, 3.14)
Nongovernment employee	3.32 (1.48, 7.45)	3.72 (1.58, 8.76)	3.08 (1.31, 7.26)	0.62 (0.44, 0.86)	1.85 (1.26, 2.71)	2.00 (1.36, 2.96)	0.80 (0.64, 1.00)	1.57 (1.23, 2.01)	1.58 (1.24, 2.01)	1.42 (0.06, 2.10)	2.35 (1.51, 3.66)	2.50 (1.61, 3.89)
Government employee	6.04 (2.60, 14.01)	4.89 (2.03, 11.79)	3.27 (1.34, 7.99)	0.26 (0.17, 0.40)	0.54 (0.33, 0.87)	1.41 (0.85, 2.32)	0.42 (0.32, 0.54)	0.72 (0.54, 0.97)	1.36 (1.02, 1.82)	0.64 (0.39, 1.05)	0.82 (0.48, 1.39)	1.74 (1.02, 2.97)

Model 1: unadjusted estimates. Model 2: adjusted for age, gender, area of residence, and geographic region. Model 3: adjusted for age, gender, area of residence, geographic region, and other socioeconomic variables.
